# How the service delivery works in the Iranian specialised burns hospitals? A qualitative approach

**DOI:** 10.1371/journal.pone.0216489

**Published:** 2019-05-21

**Authors:** Nasrin Shaarbafchi Zadeh, Farzaneh Mohammadi, Mostafa Amini Rarani, Marzieh Javadi, Mohammadjavad Mohammadzade, Vahid Yazdi-Feyzabadi

**Affiliations:** 1 Social determinants of Health Research Center, Isfahan University of Medical Sciences, Isfahan, Iran; 2 Health Management and Economics Research Center, Isfahan University of Medical Sciences, Isfahan, Iran; 3 Health Services Management Research Center, Institute for Futures Studies in Health, Kerman University of Medical Sciences, Kerman, Iran; Iran University of Medical Sciences, ISLAMIC REPUBLIC OF IRAN

## Abstract

As burn injuries are a major cause of death and infirmity, successful service delivery is vital in health systems. In Iran, a few specialised burns hospitals (SBHs) located in big provinces provide burn services in which burn patients with more severe conditions are referred to. However, SBHs are faced with several challenges for delivering due treatment for burn patients. So, for the first time in Iran, the main aim of the study was to identify the challenges of delivering burn services in SBHs. For this purpose, we conducted a qualitative study during February 2017 to April 2018. Key informants were purposefully selected and interviewed at national and provincial levels from the Ministry of Health, medical universities, and informants working in eight SBHs. The saturation point was reached at 21 face-to-face semi-structured interviews. A thematic analysis approach was employed to analyse transcribed documents assisted by MAXQDA Plus version 12. Our results reveal four themes and twelve subthemes on the challenges of delivering services in SBHs. Themes and (subthemes) including burn care continuum (preventive care, pre-hospital care, hospital care, follow-up, and home care), regionalisation of burning services (access to other specialties and medical services, access to specialized care in provinces without a SBH, standardised regionalisation system for burn related services (BRSs), costs of providing BRSs (expensive services and supplies and long hospitalisation), and non-compliance with standardised care (guidelines to provide burn care and physical space to provide BRSs). Results suggest that improving BRSs delivery in Iran may be reached by strengthening burn care continuum, regionalising burn care, allocating sufficient budgets to burn services and formulating burn care guidelines. These policy actions can be better addressed via intra-sectoral collaborations.

## Introduction

Health service delivery is an essential element of any health system [1]. The goal of health delivery is to improve the health of the community and respond to the expectations of the people, together with inequality reduction in both cases [2]. In burn injuries, successful service delivery is very important because burn injuries are a major cause of death and infirmity around the world. It is estimated that more than 300,000 people die every year from fire-related burn injuries. Millions suffer from burn injuries, many of which are permanent [3]. Burn injuries result in the imposition of short-term and long-term costs to the victim, families, communities and nations [4]. The mortality rate of burn injuries varies in different parts of the world. Progress in the treatment and care of burn patients has also contributed to reducing the mortality rate in many high-income countries [3]. Burning care costs up to $1000 a day per patient in developed countries [5]. But, in the majority of low and middle-income countries, there is a lack of major surgical infrastructure such as an operating room, high tech instruments and a lack of skilled burn medical and nurse personnel. In addition, they experience an unpredictable supply chain constraints on raw materials and wound dressing [6]. Three WHO regions including Eastern Mediterranean, Southeast Asia and Africa account for almost two third of the total burden of injuries [7].

In Iran, the 2003 global burden of disease study showed that burn injury is the 13th cause of the burden of disease in the general population and the seventh cause in children aged 5 to 14 years old [8]. Trends in burn incidence showed that an increase of burn admissions or burn incidence rates in Iran between 2005–2009 [9]. Moreover, the rate of burn-related mortality was reported 3.8 for every 100 thousand individuals in Iran [10].

The treatment of burns is complex, expensive and time-consuming, because patients need special care, well-equipped medical instruments and trained staff [8]. Currently, the burn delivery system is not clearly defined in Iran’s health system. A small number of specialised burns hospitals (SBHs) located in big provinces provide burn services in which burn patients with more severe conditions are referred to them. Simultaneously, burn units in general hospitals, also, provide primary burn care services.

Since 2013, special measures have been taken to treat burn patients in the form of the Health Evaluation Plan in Iran, including the development of specialised burns sections and hospital emergency equipment, the development and standardisation of burn wards and burn medical care units (BICUs). As more burns occurred in the poor, vulnerable communities and underprivileged districts, treatment deputy of Iran’s Ministry of Health also prioritising burn cares in order to provide fair access to essential burn services for all through scaling up equitable access to burn cares in the society.

Despite the numerous advances, the gap between effective action and demand remains remarkable. Having money and technology is not sufficient for efficiency. Even with more money and better technologies, there remains a major challenge: improving health services [11]. In such a situation, identifying the challenges for delivering burn services is an invaluable field of study. To the best of our knowledge, no comprehensive studies were found in this field in Iran. Nonetheless, in South Asia, a lack of resources at current burn centres, lack of specialised care facilities, overcrowding of centres, delay in follow-up care for burns, lack of funding for low-income and people living in rural areas, and the lack of specialised rehabilitation services for burns were major challenges [12].

Jagnoor et al. (2018) identified a number of barriers to providing burn care services in India. This study showed that the lack of operating standards for burn treatment, different knowledge and skills from health professionals in providing burn care, along with resource shortages, affect the quality of burn care for patients [13]. Jagnoor et al. (2017) identified the challenges of burn and recovery care such as poor communication between health care providers and burn victims, limited rehabilitation services, transportation problems to health centres, and high costs of burn treatment and confirmed the need for long-term rehabilitation [14]. Wang et al. (2018) identified the challenges and improvements in burn wound healing, infection, pain and wound and explained there are challenges in long-term treatment and wound healing, infection, pain and hypertrophic wounds in burn management [15].

Given the importance of the delivering burn services for due treatment of burn patients as well as the significance of burn services for burn victims and their families, the aim of this study was to identify the challenges of delivering burn services in SBHs in Iran. This study will inform policymakers and health authorities to adopt appropriate policy for providing burn services.

## Materials and methods

This study received the required ethics approval from Isfahan University of Medical Sciences Research Ethics Committee, Isfahan, Iran with ethics code No. IR.MUI.REC.1395.2.292. Moreover, participants’ informed consent was acquired from all study participant and assured their anonymity and confidentiality of any information they might present.

A qualitative study using content analysis approach was designed to investigate the challenges of service delivery in specialised burns hospitals. The study was performed during February 2017 to April 2018 at the national level of the country. The population of study include all key informants related to the burn services delivery in Iran. To identify and select key informants related to the phenomenon of interest, purposeful sampling technique with a maximum variation approach was used. Maximum variation allows us to explore important shared patterns that cut across heterogeneities. The informant participants were recruited at national and provincial levels from Ministry of Health and Medical Education (MoHME) and medical university experts. Also, CEOs, managers, dermatologists, matrons and supervisor nurses that have worked in cities owning SBHs (Tehran, Isfahan, Ahvaz, Shiraz, Bushehr, Mashhad, Tabriz and Yazd) were interviewed. Accordingly, the eligible interviewees were key informants related to burn care delivery across different provinces of Iran. Key informants were excluded for this study if they had an experience <5 years and/or cancelled the interview meeting more than four times.

The required data was gathered through face-to-face, semi-structured interviews with the participants. We developed an interview guide informed by the World Health Organisation’s framework for health system functions [1] and the research objectives (S1 File). Further, to check the validity of the interview guide, at first the questions of interview had been discussed among research team with collaboration of one external expert and revised, accordingly. Later, the interview guide was tested on three non-participants to verify the number and order of the questions in the study.

Interviews were carried out in the interviewee’s office or any places where the participant suggested by one trained member of the study team (MJM). The interviews continued until a saturation point in which no additional data collection was necessary. It is attained with the analysis and comparison of interview contents until no new or relevant data seems to be emerging concerning a theme. Lastly, 21 face-to-face semi-structured interviews were conducted. Each interview time lasted between 30–90 minutes, with an average of 40 minutes. Most interviews were tape-recorded (with participants’ informed consent) and then transcribed verbatim. One of the participants (number 10) did not allow to record his voice, so that interviewer was take field notes.

A thematic analysis with an inductive approach was employed to analysis of transcribed documents assisted by MAXQDA Plus version 12 (Release 12.3.0, VERBI GmbH Berlin). A step-by-step guide proposed by Braun and Clarke [16] was served to conducting thematic analysis. Accordingly, the following steps were done: (1) three of the authors (MAR, FM, and NSZ) as data coders, familiarised with data by immersed themselves in the data by listening to recorded interviews and reading and re-reading transcribed data. (2) the initial list of ideas behind the data was generated and initial codes from the data were produced. In this stage, the most basic segment and repeated interesting patterns were identified and related to each code. (3) after all the data were initially coded and collated, codes were analysed aiming at combining different codes to an inclusive theme. At this stage we also found out sub-themes within themes, viz., essentially themes within a theme. (4) the themes were reviewed and refined during two 3-hour sessions with the main members of the research team in which all the accumulated extracts for each theme were read and coherence of their patterns were considered. (5) at this point, defining and naming of themes was done. Besides paraphrasing the themes, overlaps between themes and sub-themes and their relations to the others were examined carefully. Also, final names were given to the themes and sub-themes. (6) the report as the final opportunity for analysis was produced and scholarly publication wrote-up.

For assessing the quality of current qualitative study, we ensured four trustworthiness criteria suggested by Lincoln and Guba [17]. Credibility was ensured with a prolonged engagement (the study process continued nearly fifteen months) and respondent validation (the process whereby the researchers provided some transcribed interviews to the participants and asked them to ensure that there is a good correspondence between their findings and the perspectives of participants). Further, to improve credibility, opposite issues in findings were discussed among research team to reveal the reasons. Transferability of our qualitative findings was enhanced through purposive sampling technique and thick descriptions. Dependability of the research was adopted by an auditing approach in which the study’s colleagues accompanying by an external auditor engaged in complementary comments in coding process and analysing of interview text as well as cross-checked the data we collected. To increase confirmability, we not openly allowed our personal values to conduct the research and the findings obtaining from it. According to the Hewitt-Taylor [18], we maintained reflexivity by abstractly hypothesizing in a more divorced manner concerning research questions and how the data we gathered gave insight into these, as well we have used field note taking to enrich the data. Moreover, we have tried to shrink the impact of our experiences on different stages of the study process [19].

## Results

Interviewees are described in Table 1 (i.e. their organisation affiliation, years of experience and city).

**Table 1:** Characteristics of participants.

participant	Organisation	City	Experience
1	Specialised burns hospital	Isfahan	26
2	Specialised burns hospital	Isfahan	15
3	MoHME	Tehran	18
4	Medical University	Isfahan	17
5	Specialised burns hospital	Ahvaz	10
6	Medical University	Isfahan	9
7	Specialised burns hospital	Shiraz	12
8	Specialised burns hospital	Shiraz	22
9	Specialised burns hospital	Bushehr	13
10	Specialised burns hospital	Tehran	17
11	Medical University	Tehran	23
12	Specialised burns hospital	Mashhad	19
13	Medical University	Mashhad	8
14	Specialised burns hospital	Mashhad	15
15	Medical University	Ahvaz	16
16	Specialised burns hospital	Tabriz	11
17	Specialised burns hospital	Tabriz	8
18	Medical University	Tabriz	6
19	Specialised burns hospital	Yazd	10
20	Specialised burns hospital	Yazd	14
21	MoHME	Tehran	23

Four themes and twelve subthemes on challenges of delivering services in SBHs were extracted. Themes related to challenges of SBHs were as follows: burn care continuum, regionalisation of burning services, costs of providing BRSs, and non-compliance with standardized care (Table 2 and S1 Fig).

**Table 2:** Themes, sub-themes, and codes related to the challenges of delivering services at SBHs.

Themes	Sub- themes	Codes
Burn care continuum	Preventive care	- Peoples’ inadequate knowledge about burn prevention - Lack of a national prevention plan - Lack of advocacy and participation of all society sectors in burn prevention - Inadequate media activities to train burn prevention
	Pre-hospital care	- Physicians insufficient skill in providing pre-hospital care in non-specialised hospitals - Insufficient skills of emergency medical technicians to provide first aid care - Inadequate equipped ambulances - Delays in sending patient to BSHs
	Hospital care	- Poor access to specialised health professionals to provide BRSs - Deformity and pastiness after treatment - Lack of collaboration of patient companion(s) to control infection and to maintain isolation standards - Ignoring emotional and mental needs of patients - Inadequate provision of high calorie and protein nutrition - Not using latest available treatments.
	Treatment follow-up	- Lack of patients’ active follow-up system - Lack of after discharge care - Financial barriers for after discharge follow-ups
	Home care	- Lack of strengthened home care service - Insufficient skilled nurses to provide home care services - Failure to observe patients and their companion’s obedience to burn treatment
Regionalisation of BRSs	Poor access to other specialties and medical services	- Insufficient provision of healthcare services to multi-trauma burn patients and cases with co-morbidity - Lack of a specialised teams - Poor access to advanced diagnostic and laboratory services
	Insufficient access to specialised care in provinces without a BSH	- Limitations in transporting patients due to long distance, lack of standardised transportation and costs of transportation - Problems related to hoteling - Delay in admission or not admitting due to shortage of beds in BSHs - Lack of specialised equipment and medicines
	Lack of a defined and standardised regionalisation system for BRSs	- Lack of official regionalisation system - Referring patients without referral letter - Separation of BSHs from other hospital centres - Ignoring regionalization standards in BSHs - Lack of defined protocols to burn patients’ triage
Costs related to the burn service provision	Expensive services and supplies	- Expensive treatment - High costs of service provision
	Costs of long hospitalization in SBHs	- Increased workload of SBHs - Increased bed occupation rate - Increased costs of treating patients
Non-compliance with standardised care	lack of guidelines to provide burn care	- Lack of defined guideline to care different burned organs - Lack of clear guideline to control infections of burn wounds - Applying personal opinions to different stages of treatment
	Unstandardised physical space to provide BRSs	- Inadequate physical space for hospitalisation - Ignoring standards of burn beds per population - Ignoring standards of intensive care beds for burn patients per population

### Burn care continuum

#### Preventive care

Given that a burn does not have a definitive treatment and is a chronic disease, the best way to treat it is prevention. However, there is no a National Program on Burn Prevention (NPBP). One of the interviewees noted that:

“*I emphasise that the best treatment for burn is prevention*, *prevention*, *and prevention*. *And the best strategy is to develop a NPBP*, *that must be developed by the MoHME” (interviewee number 2)*.

The necessity of developing regulations on burn prevention by the MoHME as well as advocacy of ministries of other sectors, particularly the Ministry of Petroleum and the Ministry of Energy, and their engagement in developing a NPBP are among important issues noted by interviewees. Inadequate training of different groups of the society is an important challenge to provide preventive care, particularly given that most of the burns occur in individuals with lower levels of literacy. As an interviewee acknowledged, preventing the burn is better than providing treatment, as prevention is better than treatment. Appropriate and adequate training can substantially reduce burn probability:

“*On my opinion*, *training*, *training and training are the key to prevent burn*. *Most of the burns are preventable*, *if primary trainings be available for households*, *at workplace and society level … prevention of burn is better than treatment” (interviewee number 9)*.

Interviewees mentioned the insufficient awareness about burn prevention and burn patients as well as insufficient attention of media, particularly the national TV, for training of burn prevention.

#### Pre-hospital care

Pre-hospital measures, such as fluid therapy, oxygen therapy, catheterisation, and early bandaging, are vital. These measures prevent expansion of burn wound, renal complications, shock, damage to cells, and complication of treatment process. However, based on interviewees’ responses there are some challenges as follow: insufficient skills of physicians in providing pre-hospital care in non-specialized centres, insufficient skills of emergency medical technicians in providing first aid cares to burn patients, shortage of ambulance to transport patients, and delays in referring patients to SBHs:

“*Burn patients first need first aid*, *which if not provided*, *can results in complications*. *This results in delayed treatment or serious damage to cells*, *which is not treatable and even can led into death*. *One of the best strategies that the MoHME can take is implementing widespread trainings in non-specialised centres*, *hence most of the patients can receive effective treatment at the first available healthcare centre*. *Unfortunately*, *appropriate services are not providing to burn patients*. *There is no trained team and equipped ambulance*, *and sometimes due to late referral*, *we can't do much” (interviewee number 19)*.

#### Hospital care

Challenges to provide hospital care are one of the important challenges in providing BRSs. As one of the interviewees noted, the treatment structure of SBHs led into either patients’ death or discharging them with deformity and sticky wounds. Many of these consequences remain for the rest of life, even after several surgeries. Failure to observe infection control codes by patients’ companion(s) is another challenge. Companion(s) must observe all sterile and isolation standards. As well, there are limitations and regulations that must be observed by companion(s), which are not always observed. Another challenge is lack or inadequacy of psychologist in SBHs to meet psychological needs of patients.

For patients’ nutrition, on one hand the SBHs have problems in providing high calorie and protein food regimens, and on the other hand, families can’t afford the costs of appropriate food regimens. Insufficient access to health professionals is another problem of patients with burn problems. Due to a low number of physicians in evening and night shifts, and high workloads, physicians do not spend enough time for patients. One supervisor nurse told:

“*In terms of accessibility … unfortunately access of burn patients to health professionals is too low*. *For example*, *our hospital only has one physician*. *In an ideal situation*, *only spends one minute for each patient…these are only on the paper*, *real world is different” (interviewee number 17)*.

As well, sometimes general practitioners examine burn patients, that don’t provide burn therapy. As most of interviewees noted, burn patients do not receive advanced and new therapies:

“*When we look at available therapies*, *still most of them are traditional*, *I mean for three weeks we only wash wounds*, *use ointment and disinfectants*, *and in some cases new bandages are used*, *but in practice three weeks must pass to see whether the wound is type 2 or type 3*, *if it is type 2*, *the wounds are healed*, *and if it is type 3*, *debridement*, *grafting*, *and early oxygen will be used” (interviewee number 2)*.

#### Follow up

For patients, a burn is a chronic disease. Most of the treatments must be provided after patients’ discharge. When patient is discharged, must be followed by health professional to ensure that necessary care, nutritional regimen, dressing codes, and rehabilitations services as well as social-spiritual supports are received and observed, to avoid sticky wounds, deformities, scars, and other consequences. As one of the interviewees mentioned, lack of active follow-up system for to monitor after patients’ services is a serious challenge. Treatment can be provided through referral system along with active role of family physician. However, financial barriers are mentioned as an important factor. A national policy-maker noted that:

“*The problem is that patients are not followed after discharge*, *and can't afford further treatment costs*. *Acute phase is passed*, *but due to financial barriers*, *patients don't follow after discharge treatment orders*. *Patients never back*, *because costs are too high*. *Referral of burn patients is between 30 to 40%*, *but referral for burn consequences is almost zero*. *Few patients refer to healthcare centres to treat sticky wounds or deformities*, *while their prevalence is high*” *(interviewee number 3)*.

#### Home care

University professionals note that developed countries are making policies to reduce hospitalisation, particularly for lower degree burns, that can be treated at home. Home care services can reduce hospital expenditures, increase participation of households in patients' treatment, and reduce hospital infections. Challenges related to the home care are as follow: lack of strengthened home care services at SBHs, an insufficient number of nurses to provide home care services, difficulties of providing home care services to referred patients from other cities and towns, and ignoring obedience of home care treatment by patients and their companions.

### Burn services regionalisation

#### Poor access to other specialties and medical services

Inadequate access to other specialties in SBHs is an important challenge to provide BRSs in Iran. Given that in burn many organs are damaged and patients are typically multi-trauma (e.g. burn and falling due to electrocution, traffic-related accident and burn, and acid burn with eye damages), due to lack of all specialties in SBHs, and of course a lack of economic justification, patients' treatment faces with barriers. In such cases patients must be referred to other healthcare centres or healthcare professional from other centres must be invited to join the treatment team, that each has its own problems and challenges, such as patients' immobility and delays in service provision. In addition, in some cases, burn patients have co-morbidities, such as diabetes, cardiovascular diseases, etc, or a pregnant woman may face a burn. In such cases, services cannot be provided appropriately. An interviewee noted that: *“Given that it’s a single specialty hospital and is only for burn patients*, *it has the advantage of easier control of infection and treating wounds (i*.*e*. *the probability of infection transmission is lower)*, *but given that burn patients need multiply specialties there are problems in access to healthcare services*. *In such cases health professionals must be invited from other centres or patients must be referred*, *which each has its own challenges*, *and typically all needed services won't be received (interviewee number 7)*.

An interviewee responded that: *“Service provision for burn patients requires team work (SBHs must provide multidimensional services*, *i*.*e*. *physiotherapy*, *nutrition*, *anaesthesia*, *internal medicine*, *surgery*, *eye*, *CVD*, *brain*, *liquid therapy and etc*.*)*, *it means that a team must be formed” (interviewee number 12)*.

The SBHs can't have multi-speciality teams, which is against current standards of treating burn patients. Complementary diagnostic tests are another challenge. Due to a lack of advanced laboratories, the SBHs typically sign contracts with other hospitals to receive advanced laboratory services, which creates problems for coordination, in time admission, and, sometimes, reluctance to accept burn patients due to their physical status (deformity and obnoxiousness).

#### Inadequate access to specialised care in provinces without a SBH

Specialised care for burn patients are only available in eight provinces (out of 31), that has led into challenges in provinces without a SBH. For most of the provinces, the distance between province capitals is long, then patients’ conditions got worse and even may die. Sometimes, SBHs’ beds are fully occupied and patients cannot be hospitalised, or it may be delayed. Transportation costs and problems related to hoteling services for burn patients are among other challenges that are mentioned in the current study. One of the interviewees noted that: “*Transporting patients from general hospitals to a SBH usually creates problems*. *There are costs that must be paid to transport the patient*. *Even if we ignore treatment costs*, *companion(s) needs a place to stay*, *which has its own costs*. *Who pays the costs*? *The companion(s) does not have enough money*. *Even we have staffs in our hospital which raise money to buy food and fruits for companions*. *Or there are companion(s) who takes the hospital foods and eat in streets around the hospital” (interviewee number 10)*.

A lack of trained and skilled staff as well as shortage of equipment and medicines for burn patients in cities without a SBH, are among the other mentioned challenges that can result in hospital infections, infection of burn wounds, increased mortality, and deformity.

#### Lack of a defined and standardized regionalization system for BRSs

Based on interviewees’ responses, currently the Iranian healthcare system doesn’t have a defined and appropriate service regionalisation for BRSs. Although during the past years, measures are performed to address the shortcomings, but they were not enough. However, there may be informal contracts between SBHs to referrer patients, but there is no formal system. A policy-maker engaged in policies related to burn patients noted that:

“*There are issues*: *first*, *we don’t have a service regionalisation system*. *Currently*, *patients can be referred from smaller hospitals to larger*, *or from smaller to larger provinces*, *but still we don’t have a defined system*, *at least I think so*. *Because*, *to employ a defined system*, *some hospital wards and services must be considered*, *then it should be determined that which services must be provided at what levels*, *and*, *also*, *the referral chain must be defined*. *Based on what mentioned above*, *I think we don’t have a defined system*. *I didn’t see a report on such a system*, *but we have hospitals that are specialized to treat burn patients*, *therefore patients are referring to these hospitals*, *but it is not developed and planned” (interviewee number 21)*.

Based on the responses of many of the interviewees, current structure of providing services to burn patients is not acceptable, regarding the defined standards. Separating the SBHs from other hospital can harm burn patients’ treatment process, instead of improving the quality of services. Non-compliance with service regionalisation standards, mainly due to shortage of beds, skilled personnel, and physical space in the SBHs, is another important challenge.

Given interviewees’ responses, triaging patients based on burn degree and a defined protocol, is a neglected measure. Triaging patients into three degrees is too important: degree one, there is no need for hospitalisation, only by outpatient services patients can be treated; degree two, patients must be hospitalised in burn wards of general hospitals; and degree three, specialised services are needed and patients must be hospitalised in a SBH. Therefore, the next challenge is patients’ triage. One of the interviewees noted that: *“Triaging patients is the first step of the burn patients’ treatment*, *particularly if many patients are damaged*. *For example*, *imagine that a big building is on fire and many are burned*, *the first step is to triage patients*, *i*.*e*. *the percentage of burn injury and its depth must be determined*. *Then*, *based on the percentage area of burn and its depth*, *patients must be categorized” (interviewee number 6)*.

### Costs of burn service provision

#### Expensive services and supplies

Expensive services and supplies are an important challenge to provide BRSs. It is said that burn is expensive, which creates problems at both demand and prevention sides. For the demand side, due to the lack of financial ability or being poor many patients do not seek treatment or, if seek, don’t receive complete services, due to high price of medicines and surgeries. Generally, burn is an additional painful tragedy for many patients. One of the interviewees noted that: “*There are patients who cannot afford the treatment costs … for about 80% of them*, *the burn is an additional bad tragedy*, *because they are poor and cannot pay the bills … secondly*, *if they can afford the costs*, *medicines are too expensive and numerous*, *so that the family faces catastrophic payments” (interviewee number 12)*.

On the supply side, because of using new bandages, antibiotics, strong disinfectants, expensive supplies, plastic surgeries (including skin grafting), and high depreciation of equipment, for most of the SBHs costs are higher than incomes and allocated budgets. Hence, they face severe challenges to provide BRSs:

“*Plastic surgeries have a substantial role in expensiveness of BRSs*, *as well there are new bandages and medicines*, *such as Nano-bandages*, *that are too expensive*, *of course are more effective than traditional bandages*. *As the time passes*, *the prevalence of antibiotic resistance increases*, *then stronger antibiotics must be used*, *which in turn increase the treatment costs*. *Therefore*, *new bandages and surgeries increase the treatment cost” (interviewee number 17)*.

#### Costs of long hospitalisation in SBHs

Long hospitalisation is an important challenge that increases the treatment costs for burn patients:

“*Burns are costly*, *are different that an appendicitis surgery which patient can be discharged in the day after surgery*. *I mean most of burn patients are hospitalized for more than two weeks*, *then there are problems in service provision” (interviewee number 17)*.

Usually, long hospitalisation both increases the treatment costs, and hospitals' workload and bed occupancy ratio. At the same time, beds turnover is low. These factors increase the costs of SBHs in comparison to other specialised hospitals.

### Non-compliance with standardised care

#### Lack of guidelines to provide care

Lack of guidelines to provide care for burn-related patients is another important challenge that has created problems. If guidelines be available, service provision can be facilitated. For example, what services should be provided in cases of acid burn or gas or fire. Also, treatment of wound injuries, how to move burn patients, and how to care different organs that are burned can be defined. Currently, due to lack of guidelines there is no clear instruction to treat burn patients, and personal decisions have an important role that sometimes can be non-scientific. One of the interviewees noted that:

“*We don’t have a standard to treat burn patients*, *there is no guideline at all*. *An important problem of the country is lack of instructions to treat patients*. *We don’t have a role*, *providers treat patients based on their personal decisions*, *and it is the main problem” (interviewee number 4)*.

### Unstandardised physical space to provide services

Currently, in terms of physical space and number of beds, we have a lot to do to reach global standards. Indeed, insufficient physical space, in comparison with number of patients, creates challenges for treatment of burn patients. A policy-maker noted that:

“*The MoHME has developed new standards for burn-related beds*, *and also has determined the number of intensive care beds per general beds*. *Standards are too limit*. *Recently we came to the conclusion that one bed per each 30000 populations is required*, *and per each four general burn beds an intensive care bed is necessary*. *As I know*, *the standards were officially announced at March 2017*, *before that no standard was available*. *Based on the current standards*, *still there is a significant gap*. *As well*, *there is problem*, *the standard only determined that with regard to the physical space how much beds can be available*. *This is wrong” (interviewee number 17)*.

## Discussion

The current study aimed to identify challenges in providing healthcare services in SBHs in Iran, based on a qualitative approach. The results revealed that there are four themes including defects of burn care continuum, regionalization of BRSs, costs related to the burn service provision, non-compliance with standardised care.

Defects of burn care continuum are facing with a variety of challenges in continuum burn care delivery from preventive care and pre-hospital care to home care. The results showed that challenges of preventive care are related to people’s insufficient awareness, inadequate media notification, and particularly, a lack of a national plan for burn prevention. Based on the WHO report, developed countries have substantially reduced burn-related deaths during the past four decades, which most of that can be attributed to the burn prevention. Although, using advanced treatments had a substantial role in this decline [3]. Rode et al. in 2015 have also emphasised the necessity and impact of society education about burn prevention [20]. They showed that implementing prevention strategy results in significant improvement, especially in primary and secondary prevention. Also, some other studies noted that weak performance of burn wards is not due to hospital performance or severity of injuries, but it is mostly due to society’s awareness about burn, primary measures and prevention [13]. A study by Hodgins et al. has also emphasized awareness of burns [21].The study of Atiyeh et al. also showed that low level of family education is a risk factor for burns. In this study the importance of burn prevention programs has been emphasized in burn management protocols [22].

The other important challenge was related to pre-hospital care. Burn patients reside in provinces without a SBH, usually receive poor pre-hospital emergency services and necessarily refer to the SBHs that located in a few number of Iran’s provinces. This leads to some difficulties for the patients. Insufficient skill of ambulance staff and low quality of first aid services usually result in delayed arrival of patients to hospitals, severe consequences, increased hospitalisation, and higher costs for patients and health system. Moreover, physicians in non-SBHs are not fully expert in providing pre-hospital care for burn patient such as oxygen and fluid therapies. As a result, next hospital cares will be accompanied by some side effects including sticky wounds and deformity, which require additional treatments such as plastic surgery. As well as in hospital care, supportive services such as nutritional consultations are not sufficient and generally high calorie and high protein regimes are not served. In hospital care, there is other important challenges which include the provision of out date care that is related to the financial shortages and lack of appropriate training. In hospital, burn patients’ companions often do not have the required knowledge to control infections, mostly due to a low level of health literacy. Various unplanned visits and ignoring sanitary codes by patients’ families, also increase the secondary infections in hospitalised burn patients. Jagnoor et al. showed that lack of society’s awareness on basic burn services can reduce desired consequences [13].

Lack of patients’ follow-up, mainly due to financial barriers, is another import challenge to provide high quality BRSs, which in combination with a shortage of home care, results in consequences such as infection or deformity. In addition, since specialised care is only available in specialised hospitals in big cities, receiving further care can be an important obstacle for patients in small cities. In line with the findings of the current study, Jagnoor has also found that lack of patients’ follow-up is an important challenge in treating burn patients in India. Many of the identified challenges were related to the patients’ socioeconomic status, their literacy level and lack of facilities [13].

Regionalisation of BRSs was another theme in the current study, which include three themes. First, poor access to other specialities and medical services. Due to deliver specialised burn services only in SBHs, burn patients with multi-trauma and co-morbidity that may need cardiovascular, vision, and other specialised cares are exposed with a heavy cost burden, as well it imposes costs on the health system. In addition, this situation usually leads into delayed provision of medical services and patients’ referral to other hospitals. A lack of multi-disciplinary teams along with inadequate access to advanced diagnostic technologies are among other important obstacles to provided BRSs. Jagnoor also has mentioned that inadequate specialised and experienced human resources as important obstacles to provide BRSs. But, because in India BRSs are available in public hospitals, access limitation to specialised services were lesser. Besides, the authors found that insufficient skills of health staff, including physicians with general surgery skills, is an important obstacle to provide high quality BRSs in all healthcare centres [13]. In the study by Atyieh et al, the researchers emphasized on regionalization of burn care and linkage between primary, secondary and tertiary levels. They suggested a registry system for burn services [22]. Also Rode et al proposed a structure for regionalized burn services which include day hospital clinics, primary health care plus district hospital, regional hospital and provincial tertiary hospital [20].

Second, inadequate access to specialised services in provinces without a SBH is another important challenge. Long distances and lack of equipped ambulances, transportation costs, inadequate skills of staff in provinces without a SBH, and a lack of equipment and medicine along with delayed referral due to lack of burn-specialized beds, hinder the provision of high quality services. In many cases the provided services by healthcare facilities were not appropriate, that resulted into consequences such as secondary infections. In addition, delayed referral of patients to equipped healthcare centres increases the treatment period and costs. Gallaher et al. showed that in Africa BRSs are available in the burn department of general hospitals, rather than a SBH, so there are less challenges [6]. Meanwhile, Jagnoor only mentioned to the shortage of equipment and facilities in public sector, including hospital beds, to isolate patients to avoid infection [13]. In India, also, because BRSs are providing through public hospitals, there are less challenges.

Third, a lack of a defined regionalisation system for BRSs is another important challenge. Ignoring regionalisation standards and referral system sometimes lead into refusing to hospitalise patients or delayed hospitalisation, that both can result in severe consequences. Jagnoor et al.’s work is consistent with the results of current study [13]. Holmes et al. investigated the effectiveness of regionalisation from 2000 to 2007, and emphasised on the effectiveness of regionalization of BRSs. They noted that a single healthcare centre cannot provide the best possible services [23]. Literature on standardising care in SBHs show that 24 hours a day and seven days a week access to a specialised team (i.e. physicians, nurses, and other staff) to provide BRSs as well as patients’ follow-up are among basic standards. To achieve this standard, there must be a precise estimation of minimum level of staff [24]. Moreover, studies indicated that patients with major burn injury require a multi-disciplinary team. In this line, services regionalisation can be a useful step [25].

The results also showed that high costs are an important challenge. On one hand, due to the high workload of personnel, and high price of medical equipment and medicine, the cost of provided services is high. On the other hand, public resources are not sufficient to cover these costs. In a study on analysing costs of a burn ward in Africa, high cost was mention as a main challenge. This study has emphasised on possibility of provision of comprehensive BRSs with lower costs in low-and-middle income countries [6].

Finally, another challenge of BRSs’ provision in Iran is non-compliance with international standards. It can be attributed to a lack of clinical guidelines to treat different burned organs, infection control, and using personal ideas in treatment process of burn patients. In addition, defining specific standards on beds per population, intensive care unit beds per burn-related beds, physical and human resources in burn wards had intensified the gap. As interviewees noted, non-compliance with standards had resulted in low quality services. However, non-compliance is mostly the result of financial shortages in low-and-middle income countries [5]. In India, also, standards are not fully complied in which provided interventions and services substantially vary from one hospital to another. Partly it is due to lack of clinical guidelines [14].

### Limitations

There are some limitations in the current study that are worth declaring. The first limitation could be the issue of subjectivity. Nevertheless, we analysed the data by three colleagues and checked the analyses with an external reviewer to enhance credibility of our results, our interpretation of data may remain subjective and our findings cannot claim a total truth. However, given that our research theoretical paradigm is a constructive approach rather a positivism one, this situation is unavoidable and is defensible. The second possible limitation of this study is that interviewees might have been affected by a social desirability bias. Namely, interviewees may have narrated what they thought interviewers want to hear, rather than the actual events. We minimised this limitation via asking probing questions during the interview process.

## Conclusion

The current study showed that Iran has faced with several challenges in terms of delivering BRSs. The results may provide policy actions on improving of BRSs delivery, which include strengthening burn care continuum, burn care regionalisation, allocating sufficient budgets to burn services and formulating burn guidelines. As well, to provide appropriate burn services, intra-sectoral collaborations among MoHME and other organisations and institutions as well as advocacy can be helpful.

## Supporting information

S1 File Supporting material. Interview guide used for collecting qualitative data related to service delivery in the Iranian specialised burns hospitals.
(PDF)

S2 File Dataset. (MX12)

S1 Fig Frequency of themes stated by participants. (PDF)
