# Supplementary material for: How the service delivery works in the Iranian specialised burns hospitals? A qualitative approach
Source: PLoS One. 2019 May 21;14(5):e0216489. doi: 10.1371/journal.pone.0216489 (PMC6528987; doi:10.1371/journal.pone.0216489)
Supplement: S1 File — Interview guide used for collecting qualitative data related to service delivery in the Iranian specialised burns hospitals. (PDF) [file pone.0216489.s001.pdf]

Supporting Material

Interview guide for collecting qualitative data related to service delivery in the Iranian specialised burns hospitals.

Main questions	Secondary and probing questions
1- What's your idea about the current regionalisation system related to burn services delivery in Iran?	1-1- What are challenges and opportunities of burn services deliver in terms of providing services from less specialised cares to high specialised ones?<br>1-2- Is it lead to save or waste burn care expenditures?
2- What do you think about the status of delivering services in specialised burns hospitals for burn patient?	1-2- From access point of view<br>2-2- From need-based cares perspective
3- What are the effects of delivering specialised burn services in a few provinces on burn services delivery in provinces without specialised burns hospitals?	1-3- From access point of view<br>2-3- From quality point of view<br>3-3- From issues related to referral system
4- What's your opinion about the current procedures of burn cares in specialised burns hospitals?	1-4- Is it standardized?<br>2-4- Is it evidence based?
