# Supplementary figures and images for: How the service delivery works in the Iranian specialised burns hospitals? A qualitative approach
Source: PLoS One. 2019 May 21;14(5):e0216489. doi: 10.1371/journal.pone.0216489 (PMC6528987; doi:10.1371/journal.pone.0216489)

**S1 Fig**

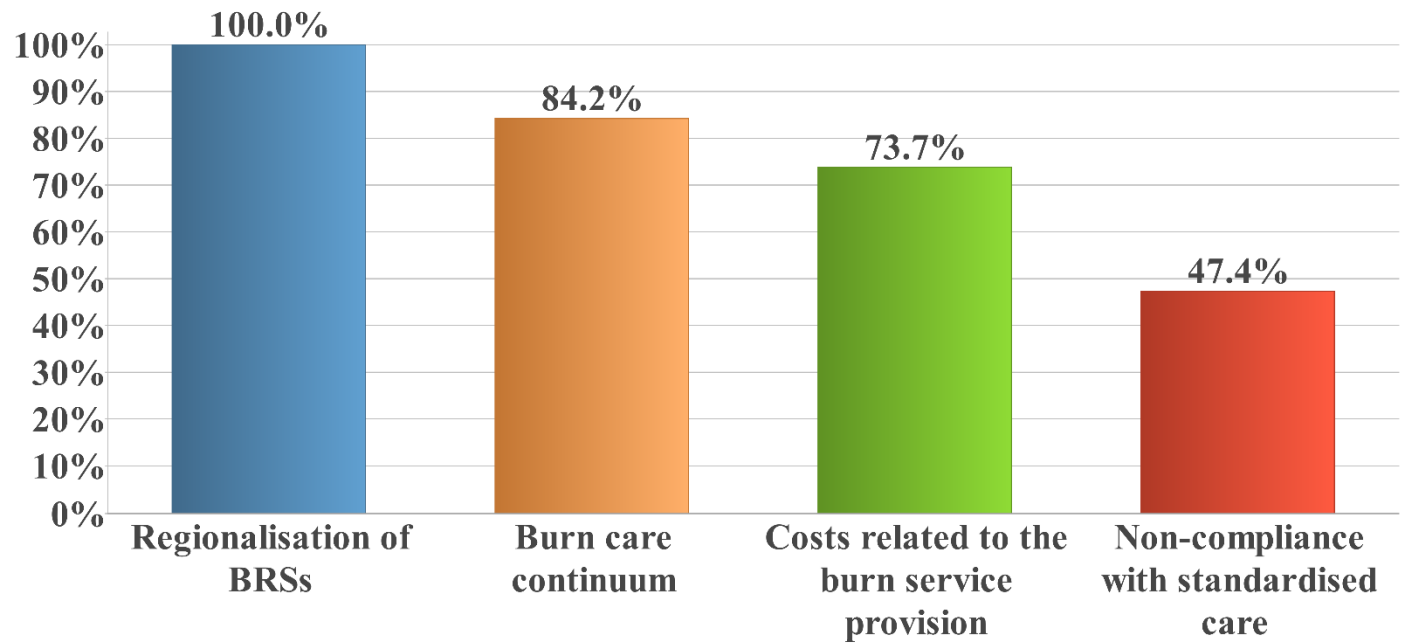

**Fig. Frequency of themes stated by participants**

Supplement: S1 Fig — (PDF) [file pone.0216489.s003.pdf]
